# Troponin I phosphorylation in human myocardium in health and disease

**DOI:** 10.1007/s12471-014-0590-4

**Published:** 2014-09-09

**Authors:** P. J. M. Wijnker, A. M. Murphy, G. J. M. Stienen, J. van der Velden

**Affiliations:** 1 Department of Physiology, Institute for Cardiovascular Research, VU University Medical Center, Van der Boechorststraat 7, 1081 BT Amsterdam, the Netherlands; 2 Department of Experimental Pharmacology and Toxicology, Cardiovascular Research Center, University Medical Center Hamburg-Eppendorf, Hamburg, Germany; 3 Department of Pediatrics/Division of Cardiology, Johns Hopkins University School of Medicine, Ross Bldg 1144/720 Rutland Avenue, Baltimore, MD 21205 USA; 4 Department of Physics and Astronomy, VU University, Amsterdam, the Netherlands; 5 ICIN-Netherlands Heart Institute, Utrecht, the Netherlands

**Keywords:** Cardiac troponin I, Phosphorylation, Myofilament function, Heart failure

## Abstract

Cardiac troponin I (cTnI) is well known as a biomarker for the diagnosis of myocardial damage. However, because of its central role in the regulation of contraction and relaxation in heart muscle, cTnI may also be a potential target for the treatment of heart failure. Studies in rodent models of cardiac disease and human heart samples showed altered phosphorylation at various sites on cTnI (i.e. site-specific phosphorylation). This is caused by altered expression and/or activity of kinases and phosphatases during heart failure development. It is not known whether these (transient) alterations in cTnI phosphorylation are beneficial or detrimental. Knowledge of the effects of site-specific cTnI phosphorylation on cardiomyocyte contractility is therefore of utmost importance for the development of new therapeutic strategies in patients with heart failure. In this review we focus on the role of cTnI phosphorylation in the healthy heart upon activation of the beta-adrenergic receptor pathway (as occurs during increased stress and exercise) and as a modulator of the Frank-Starling mechanism. Moreover, we provide an overview of recent studies which aimed to reveal the functional consequences of changes in cTnI phosphorylation in cardiac disease.

## Introduction

Cardiac troponin I (cTnI) and its binding partner cardiac troponin T (cTnT) are the biomarkers of choice for the diagnosis of myocardial damage [1, 2]. Upon myocardial injury, cTnI is degraded by calcium-sensitive proteases such as calpain I [3] and cTnI fragments are released in the blood where they can be detected via the sensitive assays that are used in practice worldwide.

**Fig. 1:**
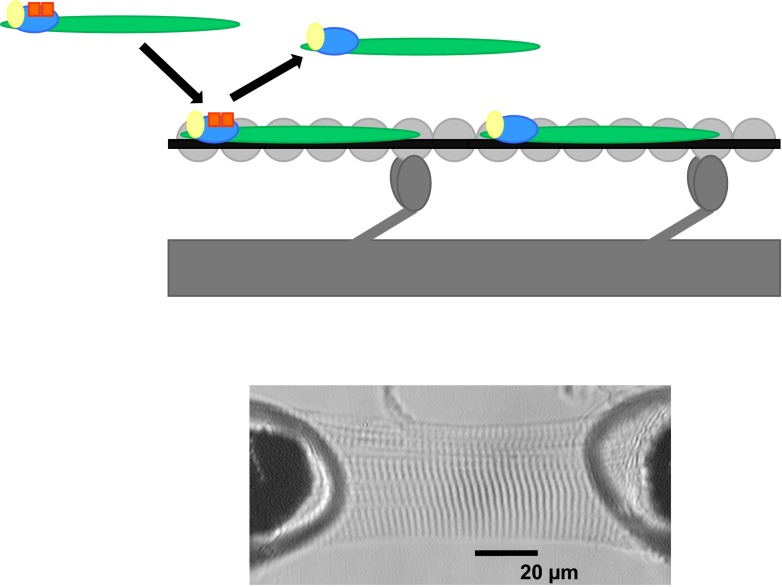
Schematic drawing of the thick and thin filaments exchanged with troponin complex. A schematic drawing of the thick and thin filaments, depicting exchange of endogenous troponin complex by exogenous troponin complex. The thick filament consists of myosin and myosin heads (*dark grey*) and the thin filament consists of actin monomers (*light grey*) spanned by tropomyosin (*black*) and the troponin complex: cTnC (*yellow*), cTnI (*blue*) and cTnT (*green*). In this drawing, exogenous cTnI is bisphosphorylated (orange squares), for example at Ser23/24. The endogenous unphosphorylated troponin complex is exchanged by exogenous phosphorylated troponin complex (*arrows*). To this end membrane-permeabilised cardiomyocytes were immersed with an exchange solution containing a high concentration of recombinant troponin complex. The lower image shows a single human cardiomyocyte in relaxing solution attached between a force transducer and a piezoelectric motor [this lower image has been published before: 18]

cTnI is an essential regulator of sarcomere contraction and relaxation. cTnI is the ‘inhibitor’ within the trimeric troponin complex, which together with cardiac troponin C (cTnC, where calcium binds) and cTnT, controls the position of tropomyosin on the thin actin filament in response to Ca^2+^ [4] (Fig. 1). In diastole (low intracellular [Ca^2+^]), cTnI binds actin at multiple sites maintaining tropomyosin at the outer domain of actin, and thereby blocks myosin-binding sites on the thin filament and prevents force development. In systole (high intracellular [Ca^2+^]), Ca^2+^ binds to cTnC and induces a conformational change in the troponin complex. This results in the release of cTnI from actin and a shift of tropomyosin closer into the grove of the actin filament, thereby enabling actin-myosin interactions and cardiomyocyte force development [5].

Because of its central role in the regulation of contraction and relaxation of the heart, cTnI may also be a potential target for treatment of heart failure. Alterations are known to occur in the phosphorylation status of cTnI during acute cardiac events and in patients with heart failure [6–8]. Studies in rodent models of cardiac disease and human heart samples showed altered phosphorylation at various sites on cTnI (i.e. site-specific phosphorylation). This is caused by altered expression and/or activity of kinases and phosphatases during the development of heart failure. It is not known whether these (transient) alterations in cTnI phosphorylation are beneficial or detrimental. Knowledge of the effects of site-specific cTnI phosphorylation on cardiomyocyte contractility is therefore of utmost importance for the development of new therapeutic strategies. In this review we focus on the role of cTnI phosphorylation in the healthy and diseased heart upon activation of the beta-adrenergic receptor pathway (as occurs during increased stress and exercise) and as a modulator of the Frank-Starling mechanism, which reflects the ability of the heart to increase stroke volume with an increase in ventricular filling (end-diastolic volume). Moreover, we provide an overview of recent studies which aimed to reveal the functional consequences of changes in cTnI phosphorylation in cardiac disease.

## cTnI-Ser23/24 phosphorylation during beta-adrenergic receptor activation

Interactions within the troponin complex are regulated by kinases and phosphatases which, respectively, phosphorylate and dephosphorylate cTnI at multiple phosphorylation sites and play a central role in tuning cardiomyocyte performance. During stress and exercise, sympathetic activation of the heart increases heart rate and stroke volume to meet the increased demands of the body. This is mediated via stimulation of β_1_-adrenergic receptors, which leads to activation of a downstream kinase, protein kinase A (PKA)[9]. PKA enhances cardiomyocyte contractility and relaxation by phosphorylation of proteins involved in Ca^2+^ handling and of myofilament proteins such as cTnI, cardiac myosin-binding protein-C (cMyBP-C), and titin (for reviews, see [9, 10]). PKA-mediated phosphorylation of Ca^2+^-handling proteins (L-type Ca^2+^ channels, phospholamban and ryanodine receptor) regulates intracellular Ca^2+^ fluxes [10], thereby controlling the amount of Ca^2+^ bound to cTnC. In addition to activated (phosphorylated) Ca^2+^-handling proteins, PKA-mediated cTnI phosphorylation at Serines 23 and 24 (Ser23/24, human sequence) represents a central mechanism controlling cardiomyocyte force development and relaxation, independent of the intracellular Ca^2+^ concentration. PKA-mediated phosphorylation at cTnI-Ser23/24 reduces myofilament Ca^2+^ sensitivity and increases the speed of relaxation of the heart which is needed to maintain cardiac performance at increased heart rates [11, 12]. Although Ser23/24 are the so-called PKA sites, multiple other kinases, PKC [13], PKD [14] and PKG [15], are known to phosphorylate cTnI at Ser23/24. The level of phosphorylation of these two serines will be determined by the balance between the kinase activity and phosphatase activity at the myofilaments, since Ser23/24 are dephosphorylated by protein phosphatases (PP) PP1 [16] and PP2A [17, 18].

## cTnI-Ser23/24 phosphorylation during beta-adrenergic receptor activation in disease

In the end-stage failing heart, PKA activity is reduced due to alterations in the β-adrenergic signalling pathway [9]. This results in decreased cTnI-Ser23/24 phosphorylation [18–21] and increased myofilament Ca^2+^ sensitivity [18, 20] in patients with ischaemic and dilated heart failure compared with non-failing donors. High myofilament Ca^2+^ sensitivity may in part underlie impaired relaxation of the diseased heart. Although cTnI-Ser23/24 bisphosphorylation has been studied extensively, effects of site-specific phosphorylation of only Ser23 or Ser24 on cardiomyocyte contractility in the human heart were unknown. Knowledge of functional consequences of monophosphorylated cTnI is important, since recent studies [21, 22] in human post-mortem control hearts and fresh donor transplant hearts with normal cardiac function revealed that approximately 40 % of cTnI is monophosphorylated. Moreover, differences in the level of monophosphorylated cTnI have been reported between donor and end-stage failing hearts [20, 21]. PKA treatment of skinned porcine cardiac muscle preparations suggested that bisphosphorylation of cTnI is required for the reduction in myofilament Ca^2+^ sensitivity [23]. However, PKA application is aspecific. We recently studied the consequences of site-specific phosphorylation at Ser23 or Ser24 in human cardiomyocytes by exchange of endogenous cTn with site-specific phosphorylated cTnI [24] (Fig. 1). This sophisticated protein engineering technique enabled us to study the effects of one specific cTnI phosphorylation site. For this purpose, phosphorylation sites (Ser23/24) in human recombinant cTnI were mutated to aspartic acid (D) to mimic phosphorylation or alanine (A), which resembles the non-phosphorylatable state. Various phosphorylated cTn complexes were introduced into human cardiomyocytes by protein exchange (Fig. 1). Using this method, we demonstrated that phosphorylation of both Ser23 and Ser24 of cTnI is required to reduce Ca^2+^ sensitivity in human cardiomyocytes, as no change in Ca^2+^ sensitivity was observed upon exchange with cTn complexes containing monophosphorylated cTnI [24]. Additionally, we demonstrated that the maximal reduction in myofilament Ca^2+^ sensitivity was reached at ~55 % bisphosphorylated cTnI [24]. Defective β-adrenergic signalling in heart failure [9] may result in a higher myofilament Ca^2+^ sensitivity when cTnI phosphorylation levels upon β-adrenergic stimulation remain below ~55 % cTnI bisphosphorylation. This may lead to an impaired diastolic function of the heart due to increased force development at low Ca^2+^ concentrations. Comparison of cardiac samples from heart failure patients with different disease severity (ranging from NYHA class I to IV) showed increased Ca^2+^ sensitivity only in the end-stage (NYHA class IV) of cardiac disease [25], which suggested that detrimental effects of reduced cTnI phosphorylation may only become evident at the end-stage of heart failure. However, a recent study in patients with obstructive hypertrophic cardiomyopathy (HCM) and normal systolic, but impaired diastolic function (NYHA class III) showed increased myofilament Ca^2+^ sensitivity [26] and lower cTnI phosphorylation [27, 28] in HCM compared with non-failing myocardium. In addition, a recent study showed that the level of cTnI bisphosphorylation in post-mortem hearts with mild hypertrophy was significantly lower (4.1 %) compared with control levels (18.4 %) [21]. Collectively, these studies indicate that cTnI bisphosphorylation and the associated impact on myofilament Ca^2+^ sensitivity depends on the stage of heart failure (NYHA class) as well as on aetiology.

## cTnI-Ser23/24 phosphorylation as a regulator of Frank-Starling mechanism in health and disease

**Fig. 2:**
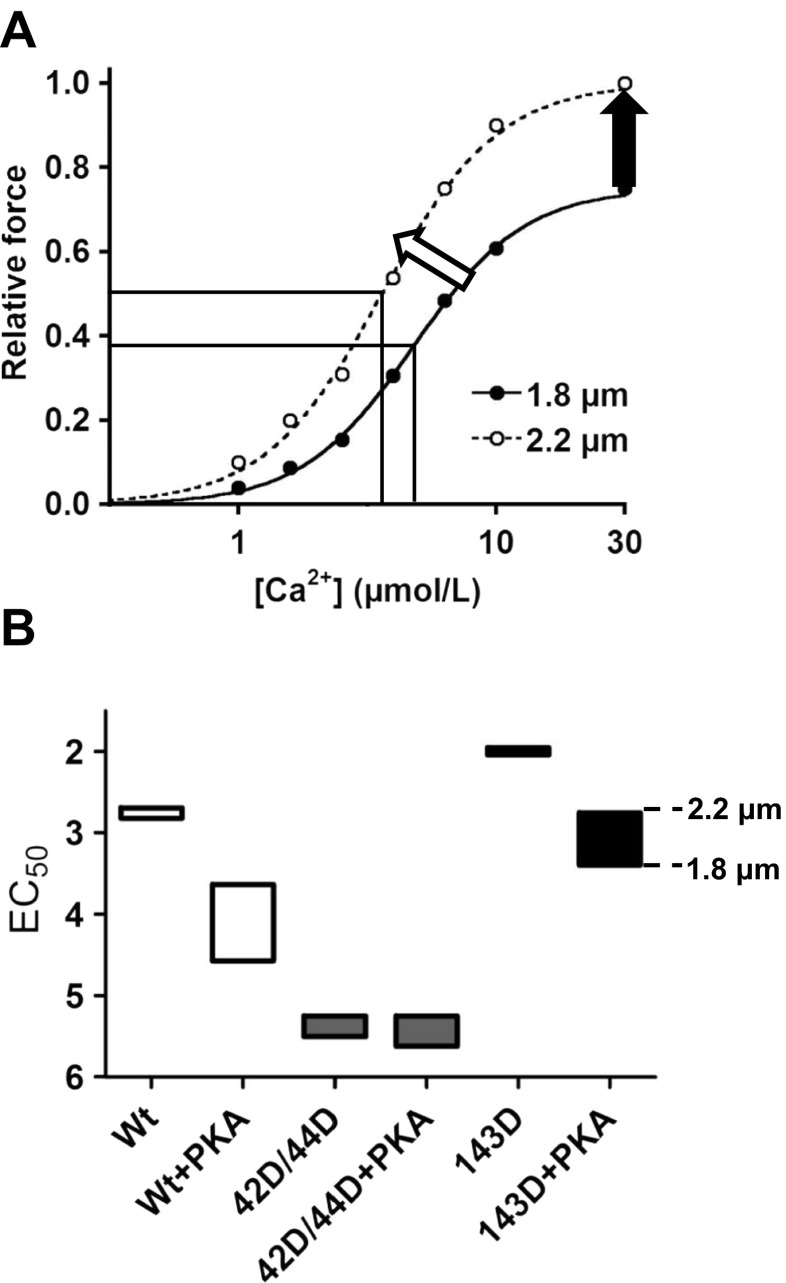
Schematic representation of changes in length-dependent Ca^2+^-sensitivity upon phosphorylation of Ser42/44, Thr143 and/or Ser23/24. **a** An example of myofilament force development at short (1.8 μm) and long (2.2 μm) sarcomere length at various [Ca^2+^] in human membrane-permeabilised cardiomyocytes. Cardiomyocyte lengthening from 1.8 μm to 2.2 μm increases myofilament Ca^2+^ sensitivity (*white arrow*). The Ca^2+^ sensitivity derived from the midpoint of the force–Ca^2+^ relationship (EC_50_) is demonstrated for both sarcomere lengths by a vertical line, and the difference represents the delta EC_50_. An increase in left ventricular filling increases myofilament Ca^2+^ sensitivity and underlies, together with an increase in maximal force-generating capacity (*black arrow*), increased cardiac output during the subsequent systolic phase. **b** Data obtained in troponin-exchanged donor cells without and with treatment with exogenous PKA [33, 41] were combined to illustrate the range at which myofilament Ca^2+^ sensitivity (EC_50_) may vary in response to phosphorylation at Ser23/24 and the PKC sites Ser42/44 and Thr143. Abbreviations: wild-type (Wt); phosphorylated 42/44 (42D/44D); phosphorylated 143 (143D). Boxes represent the range of Ca^2+^ sensitivity measured at a sarcomere length of 1.8 (*lower line*) and 2.2 μm (*upper line*). This figure demonstrates that the sarcomere length-dependent shift in Ca^2+^ sensitivity is relatively small in Wt, 42D/44D and 143D without PKA (i.e. low cTnI-Ser23/24 phosphorylation). PKA treatment of Wt and 143D increased the range in Ca^2+^ sensitivity at which the sarcomere is operating upon changes in sarcomere length between 1.8 and 2.2 μm. However, PKA treatment of 42D/44D does not enhance the length-dependent increase in Ca^2+^-sensitivity

Frank-Starling’s law reflects the ability of the heart to increase stroke volume with an increase in ventricular filling (end-diastolic volume), a property based on length-dependent activation (LDA) of the myofilaments [29, 30]. An increase in sarcomere length within the working range of the heart enhances the maximal force generating capacity and the sensitivity of the myofilaments to Ca^2+^ (Fig. 2a); i.e. for the same calcium concentration more force is generated by the sarcomeres. The contraction becomes more powerful and the ejection fraction increases. Length-dependent myofilament activation thus represents an important cellular mechanism to adjust cardiac performance in response to increased preload of the heart. The role of cTnI as a regulator of LDA of the myofilaments was demonstrated more than a decade ago via replacement of endogenous cTnI by slow skeletal troponin I (ssTnI) in cardiac muscle. This significantly blunted the increase in myofilament Ca^2+^ sensitivity upon an increase in sarcomere length [31, 32]. Furthermore, cTnI phosphorylation by PKA was demonstrated to enhance length-dependent changes in the force-Ca^2+^ relation, since PKA only enhanced LDA in non-transgenic mice, and not in a transgenic murine model where cTnI was replaced by ssTnI which misses the N-terminal region where the PKA sites Ser23/24 are located [32]. Recently, we demonstrated that PKA treatment enhanced the length-dependent increase in myofilament Ca^2+^ sensitivity in human cardiomyocytes with low levels of phosphorylated cTnI [33] (Fig. 2b: Wt vs Wt + PKA). In addition, we showed that phosphorylation at both Ser23 and Ser24 is needed to enhance the length-dependent increase in myofilament Ca^2+^ sensitivity [33]. Our data indicate that PKA-mediated cTnI-Ser23/24 phosphorylation is an important mediator of length-dependent activation in human cardiac muscle tissue.

As mentioned above, reduced cTnI-Ser23/24 phosphorylation levels have been reported in end-stage heart failure relative to explanted donor tissue [18–21]. Since cTnI-Ser23/24 phosphorylation enhances the length-dependent increase in myofilament Ca^2+^ sensitivity, reduced phosphorylation levels may reduce LDA in heart failure. This may explain why a reduced length-dependent increase in Ca^2+^ sensitivity was found in skinned fibres from terminally failing human myocardium [34]. In our study [33] replacement of endogenous cTn with exogenous bisphosphorylated cTnI-Ser23/24 enhanced the length-dependent increase in myofilament Ca^2+^ sensitivity in human failing cardiomyocytes. This suggests that the blunted length-dependent myofilament activation in end-stage heart failure [34] may be at least partly caused by low bisphosphorylation at cTnI-Ser23/24.

## cTnI phosphorylation by protein kinase C in the failing heart

While PKA activity and phosphorylation of cTnI-Ser23/24 are reduced in heart failure, it has been demonstrated that protein kinase C (PKC) isoform expression (α, β_1_, β_2_) [35] and activity [35] are increased in heart failure. Higher cTnI phosphorylation has been reported at several PKC sites [36] in failing human myocardium [6]. An increase has been found in cTnI phosphorylation at the well-known PKC sites Ser42/44 and Thr143 in end-stage failing compared with donor myocardium [6]. Although PKC-mediated phosphorylation at Ser42/44 and Thr143 and its effects on muscle contractility have been studied in vitro and in rodent models, [37] effects in humans were unknown. Therefore, we recently studied the effects of site-specific phosphorylation at Ser42/44 and Thr143 in human cardiomyocytes.

**Fig. 3:**
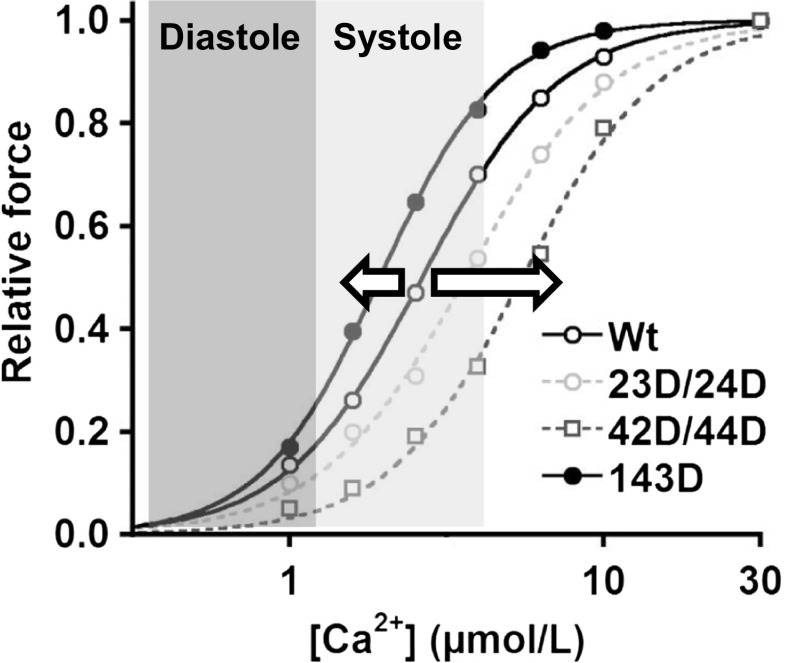
Myofilament responses to phosphorylation of cTnI at Ser23/24, Ser42/44 and Thr143 . An example of myofilament force development at 2.2 μm sarcomere length at various [Ca^2+^] in human membrane-permeabilised cardiomyocytes in which endogenous troponin complex is exchanged with exogenous recombinant troponin complexes. Compared with unphosphorylated wild-type cTnI (Wt), Ca^2+^ sensitivity (EC_50_) increases after exchange with phosphorylated cTnI at Thr143 (143D) (arrow to the left). This may result in higher cardiomyocyte force development during systole (*light grey*: high intracellular Ca^2+^ concentrations) and may result in increased cardiac output during the systolic phase, while the enhanced Ca^2+^ sensitivity may impair relaxation of the heart muscle during diastole (dark grey: low intracellular Ca^2+^ concentrations). Phosphorylation at Ser23/24 (23D/24D) or at Ser42/44 (42D/44D) decreases myofilament Ca^2+^ sensitivity compared with Wt (arrow to the right). This may contribute to enhanced muscle relaxation, which is required for proper filling of the heart during diastole, however, may result in a decrease in cardiac output during the systolic phase

Studies in rodents demonstrated that PKC-mediated phosphorylation at Ser42/44 decreases maximal force [38]. The reduction in maximal force generating capacity mediated by PKC might underlie the reduced cardiac performance observed in heart failure. However, treatment of human cardiomyocytes with the catalytic domain of PKC and different PKC isoforms (PKCα, PKCε) did not affect maximal force in non-failing donor and failing hearts [39, 40]. Our recent study in human cardiomyocytes demonstrated that replacement of endogenous cTn with exogenous bisphosphorylated cTnI-Ser42/44 induces a relatively large decrease in myofilament Ca^2+^ sensitivity (Fig. 3) without affecting maximal force development [41]. Interestingly, Ser42/44 pseudo-phosphorylation largely blunted the PKA-mediated increase in length dependence of myofilament Ca^2+^ sensitivity (Fig. 2b: 42D/44D + PKA vs WT + PKA) [41]. Since the drop in Ca^2+^ sensitivity at physiological Ca^2+^ levels was relatively large compared with phosphorylation of Ser23/24, PKC-mediated cTnI-Ser42/44 phosphorylation may result in a reduced force development *in vivo*, since the maximal intracellular Ca^2+^ concentration is ~3.98 μM [42].

Phosphorylation of cTnI-Thr143 has been reported to increase myofilament Ca^2+^ sensitivity of contraction in rodents [43]. In line with this finding, we found an increase in myofilament Ca^2+^ sensitivity in human cardiomyocytes exchanged with pseudo-phosphorylated cTnI-Thr143 [33] (Fig. 3). In addition, we studied whether phosphorylation of Thr143 affects LDA, since it has been demonstrated that the threonine residue 143 of the inhibitory region of cTnI is essential for length-dependent alterations in myofilament Ca^2+^ sensitivity [44]. Although Thr143 is essential for LDA in rodents [44], exchange with phosphorylated cTnI-Thr143 did not alter LDA (in the absence or presence of PKA) in human cardiomyocytes [33] (Fig. 2b).

## cTnI as a therapeutic target?

cTnI phosphorylation thus affects cardiac performance and its impact differs between the failing and healthy heart. Therefore, cTnI phosphorylation could be a potentially effective target for therapy. Potentially cTnI-Ser23/24 phosphorylation may be a target for correcting high myofilament Ca^2+^ sensitivity and blunted length-dependent activation in heart failure. Increased myofilament Ca^2+^ sensitivity may lead to an impaired diastolic function of the heart due to increased force development at low Ca^2+^ concentrations (Fig. 3). Also, a blunted length-dependent increase in Ca^2+^ sensitivity in end-stage heart failure has been found [33, 34], which was restored to control levels after exchange with exogenous phosphorylated Ser23/24 [33]. Length-dependent activation is the cellular basis of the Frank-Starling mechanism, which may be depressed in heart failure [45, 46]. Therefore, increasing cTnI-Ser23/24 phosphorylation in failing cardiomyocytes may be beneficial for cardiac function. This is supported by our work in human HCM samples where we showed that restoring phosphorylation at the PKA targets *in vitro* normalised myofilament function in sarcomere mutation-negative HCM and in HCM with truncating *MYBPC3* mutations [47].

The effect of PKC-mediated phosphorylation of cTnI in human depends on which sites are phosphorylated. Phosphorylation of the PKC site Thr143 did not affect LDA properties of human heart muscle (Fig. 2b), but did increase Ca^2+^ sensitivity as illustrated in Figs. 2 and 3 (Wt versus 143D, Wt + PKA versus 143D + PKA). The high myofilament Ca^2+^ sensitivity may aid to maintain cardiac output of the failing heart, however, it may also contribute to diastolic dysfunction (Fig. 3).

cTnI phosphorylation at Ser42/44 reduces Ca^2+^ sensitivity of the myofilaments and blunts the enhanced length-dependent increase in Ca^2+^ sensitivity mediated by PKA (Fig. 2b). While the low force development at physiological Ca^2+^ concentrations may reduce cardiac pump function (Fig. 3), PKC-mediated Ser42/44 phosphorylation may exert beneficial effects. The decrease in force development coincides with a decrease in ATP utilisation by the myofilaments [41], which may benefit the energy status of the failing heart. Moreover, the decrease in myofilament Ca^2+^ sensitivity may also improve diastolic function, as was suggested previously (Fig. 3) [48].

Studying cTnI phosphorylation in different disease phenotypes and at different disease stages will help us to gain a better understanding of the role of PKC-mediated cTnI phosphorylation in cardiac disease. Additionally, with this increasing knowledge about cTnI phosphorylation in cardiac disease, cTnI phosphorylation patterns at the different PKA- and PKC-phosphorylation sites may become promising biomarkers since cTnI phosphorylation status can be determined from blood samples after acute cardiac insult.
